# Looking to the future as STAM celebrates its 20th anniversary

**DOI:** 10.1080/14686996.2019.1609719

**Published:** 2019-05-20

**Authors:** Kazuhito Hashimoto

**Affiliations:** STAM Editor-in-Chief, National Institute for Materials Science, Tsukuba, Japan

*Science and Technology of Advanced Materials* (STAM) celebrates its 20th anniversary this year. The journal was launched by veteran STAM editors Tsuyoshi Masumoto and Toyonobu Yoshida in 2000 [1]. STAM was a regular subscription-based journal until 2008, when the National Institute for Materials Science (NIMS) changed its publication format to gold open access, making all STAM content free to read.

Around that time NIMS also introduced new publication policies in order to widen the areas of research covered by the journal and improve the quality of articles. Those changes resulted in the impact factor rising from 1.267 in 2008 to 4.787 in 2017 – making *STAM* the world’s leading open access journal focused on materials science. Further changes were initiated in 2016 by the former *STAM* Editor-in-Chief Shu Yamaguchi, including the launch of ‘Vision’ [2], ‘Materials Informatics’ [3] and ‘Historical Review’ [4] article categories; a materials informatics forum [5]; and an open-access publication network based at leading universities in Japan.

Since 2008 the publication of *STAM* has been managed by NIMS with the Swiss Federal Laboratories for Materials Science and Technology (Empa) joining NIMS as a *STAM* sponsor in 2016; the journal is also supported by the World Materials Research Institute Forum (WMRIF). Since 2008, the content of *STAM* has been archived in the PubMed Central (PMC) database.

Over the past 20 years, *STAM* has become internationally recognized for publishing timely, seminal articles such as reviews by Hosono et al. [6,7] and Ariga et al. [8,9]. *STAM* authors also include Nobel Prize Laureates Dan Shechtman [10], Heinrich Rohrer [11], and Ei-Ichi Negishi [12].

The publication team and editors are aware that much more work is required to enhance the journal’s visibility worldwide. Furthermore, the journal currently publishes only about 80 articles per year, so we need to increase the number of topics covered, whilst maintaining the quality of *STAM* articles.

Finally, we welcome your continued support and suggestions for publishing *STAM* as a valuable and readily accessible resource that offers critical insights into the latest discoveries in the constantly changing landscape of materials science.
